# Supplementary material for: HIVprotI: an integrated web based platform for prediction and design of HIV proteins inhibitors
Source: J Cheminform. 2018 Mar 9;10:12. doi: 10.1186/s13321-018-0266-y (PMC5845081; doi:10.1186/s13321-018-0266-y)
Supplement: Supplementary file 2 — Additional file 2. Source code of HIVProtI web server. [file 13321_2018_266_MOESM2_ESM.zip › HIVprotI_Source-code/hivprotI/weka-3-6-6/changelogs/CHANGELOG-3-5-4.html]

Weka-3-5-4 Changelog


# Weka-3-5-4 Changelog

Designed for use with Apache Ant.

---

2006-12-20 03:29 fracpete

```
updated list for GOE
```

- gui/GenericObjectEditor.props (1.115)

2006-12-19 20:22 fracpete

```
fixed NullPointerExceptions if no file extension was provided explicitely
```

- gui/ConverterFileChooser.html (1.6)

2006-12-19 20:17 fracpete

```
printClassifications didn't reset the dataset before printing the classifications (resulted in an exception)
```

- classifiers/Evaluation.html (1.71)

2006-12-19 01:54 fracpete

```
updated copyright year
```

- gui/Main.html (1.7)

2006-12-19 01:53 fracpete

```
updated copyright year
```

- gui/GUIChooser.html (1.26)

2006-12-15 03:36 fracpete

```
updated policy file
added example setup
```

- experiment/remote.policy (1.7)
- experiment/remote.policy.example (1.2)

2006-12-15 03:36 fracpete

```
added remote.policy.example file to the remoteExperimentServer.jar
```

- build.xml (1.33)

2006-12-14 04:39 fracpete

```
AllFileFilter is not used by default now
```

- gui/experiment/DatasetListPanel.html (1.22)

2006-12-14 03:46 fracpete

```
filter for adding classification, class distribution, error to a dataset based on a classifier that either got trained on the data or loaded from a serialized model
```

- filters/supervised/attribute/AddClassification.html (1.1)

2006-12-10 22:00 fracpete

```
little helper class for listing the options of an OptionHandler
```

- core/ListOptions.html (1.1)

2006-12-07 22:01 fracpete

```
added commandline generation for attribute selection scheme, in addition to the filter commandline
```

- gui/explorer/AttributeSelectionPanel.html (1.44)

2006-12-07 21:59 fracpete

```
switched to DataSource class to speed up loading for ARFF files
```

- attributeSelection/AttributeSelection.html (1.43)

2006-12-06 22:26 fracpete

```
applyFilter method outputs now the commandline string of the filter being applied
fixed some Javadoc comments
```

- gui/explorer/PreprocessPanel.html (1.66)

2006-12-06 22:26 fracpete

```
commandline option string is now output to the log
```

- gui/explorer/AssociationsPanel.html (1.23)
- gui/explorer/ClassifierPanel.html (1.100)
- gui/explorer/ClustererPanel.html (1.56)

2006-12-06 22:25 fracpete

```
added log support
commandline option string is output to log now
```

- gui/explorer/DataGeneratorPanel.html (1.3)

2006-12-05 21:50 fracpete

```
background colors now all set to "white" for a more consistent look'n'feel
```

- gui/visualize/Visualize.props (1.9)

2006-12-05 02:03 fracpete

```
popup menu popped up all the time - fixed
```

- gui/arffviewer/ArffPanel.html (1.7)

2006-12-04 23:16 fracpete

```
uses the new Databaseutils.close() method now
```

- gui/sql/QueryPanel.html (1.2)

2006-12-04 23:15 fracpete

```
added update/select methods to distinguish between queries that generate ResultSet or nothing at all
added close methods to close ResultSets and Statements in order to avoid memory leaks
```

- experiment/DatabaseResultListener.html (1.12)
- experiment/DatabaseUtils.html (1.29)
- experiment/InstanceQuery.html (1.19)

2006-12-04 23:07 fracpete

```
System.exit is now only performed if there are no more windows open and m_ExitIfNoWindowsOpen=TRUE
```

- gui/boundaryvisualizer/BoundaryVisualizer.html (1.17)

2006-12-04 23:06 fracpete

```
uses new DatabaseUtils.update/close methods instead of execute
```

- core/converters/DatabaseSaver.html (1.8)

2006-12-04 23:05 fracpete

```
JDBC URL gets updated now, in case it got changed in the User/PW dialog
cleaned up imports
removed empty main method
```

- gui/experiment/SimpleSetupPanel.html (1.12)

2006-12-04 04:46 fracpete

```
fixed getOptions - methods of superclass were added in wrong order
```

- classifiers/mi/MIBoost.html (1.5)
- classifiers/mi/MIWrapper.html (1.4)
- classifiers/mi/SimpleMI.html (1.5)

2006-12-04 04:13 fracpete

```
intial import
```

- classifiers/meta/RandomSubSpace.html (1.1)

2006-12-01 08:20 fracpete

```
added test for toString method when classifier hasn't been built yet
```

- classifiers/CheckClassifier.html (1.29)

2006-12-01 06:20 fracpete

```
setInputFormat uses now m_InputFormat instead of instanceInfo for initializing the string/relational locators - should result in smaller serialized objects, e.g., classifiers using filters internally, since the full dataset is being saved no longer (thanks to Bernhard Pfahringer).
```

- filters/Filter.html (1.33)

2006-11-29 03:58 fracpete

```
removed weka.classifiers.monotone package from list of classifier packages, since it got relocated to weka.classifiers.misc
```

- gui/GenericPropertiesCreator.props (1.12)

2006-11-29 03:56 fracpete

```
relocated from weka.classifiers.montone
```

- classifiers/misc/MinMaxExtension.html (1.1)
- classifiers/misc/OLM.html (1.1)
- classifiers/misc/OSDL.html (1.1)
- classifiers/misc/monotone/AbsoluteLossFunction.html (1.1)
- classifiers/misc/monotone/BitMatrix.html (1.1)
- classifiers/misc/monotone/BooleanBitMatrix.html (1.1)
- classifiers/misc/monotone/Coordinates.html (1.1)
- classifiers/misc/monotone/CumulativeDiscreteDistribution.html (1.1)
- classifiers/misc/monotone/DiscreteDistribution.html (1.1)
- classifiers/misc/monotone/DistributionUtils.html (1.1)
- classifiers/misc/monotone/EnumerationIterator.html (1.1)
- classifiers/misc/monotone/InstancesComparator.html (1.1)
- classifiers/misc/monotone/InstancesUtil.html (1.1)
- classifiers/misc/monotone/MultiDimensionalSort.html (1.1)
- classifiers/misc/monotone/NominalLossFunction.html (1.1)
- classifiers/misc/monotone/OSDLCore.html (1.1)
- classifiers/misc/monotone/ZeroOneLossFunction.html (1.1)

2006-11-29 03:04 fracpete

```
added the possibility to have the table in read-only mode (popup-menus are limited to the read-only options as well)
```

- gui/arffviewer/ArffSortedTableModel.html (1.3)
- gui/arffviewer/ArffTable.html (1.7)
- gui/arffviewer/ArffTableModel.html (1.7)

2006-11-29 00:46 fracpete

```
changes for PartitionedMultiFilter (needs Range to be visible in the GenericArrayEditor)
```

- gui/GUIEditors.props (1.4)

2006-11-29 00:45 fracpete

```
initial import
```

- filters/unsupervised/attribute/PartitionedMultiFilter.html (1.1)

2006-11-29 00:38 fracpete

```
InitGenericObjectEditorFilter property is now TRUE by default
```

- gui/explorer/Explorer.props (1.3)

2006-11-29 00:30 fracpete

```
uses only Swing components now - this fixed weird display errors from within the weka.gui.Main class
```

- gui/SimpleCLIPanel.html (1.2)

2006-11-26 22:00 fracpete

```
added context-menu to algorithmlist panel for copy/paste of string configurations (just like in PropertyPanel)
```

- gui/experiment/AlgorithmListPanel.html (1.18)

2006-11-26 21:35 fracpete

```
fixed NullPointerException in toString() method if no model built yet
```

- classifiers/functions/IsotonicRegression.html (1.4)

2006-11-26 20:40 fracpete

```
renamed XMLLoader/Saver to XRFFLoader/Saver
```

- core/converters/ConverterUtils.html (1.10)
- core/converters/XMLLoader.html (1.2)
- core/converters/XMLSaver.html (1.3)
- core/converters/XRFFLoader.html (1.1)
- core/converters/XRFFSaver.html (1.1)

2006-11-26 02:43 fracpete

```
in all text nodes the content is now turned into valid content, i.e., < and > get turned into &lt; and &gt; - was missing for the labels of an attribute
```

- core/xml/XMLInstances.html (1.2)

2006-11-24 00:40 mhall

```
Fixed bug that caused array out of bounds when producing a ranked list of attributes from a backwards search.
```

- attributeSelection/GreedyStepwise.html (1.8)

2006-11-22 20:40 fracpete

```
the "copy(string)" method for copying while renaming an attribute is now public
```

- core/Attribute.html (1.42)

2006-11-21 01:46 mhall

```
Default clusterer icons courtesy of Jeffrey (they were on his CD but I didn't realize it :-))
```

- gui/beans/icons/DefaultClusterer.gif (1.2)
- gui/beans/icons/DefaultClusterer\_animated.gif (1.2)

2006-11-17 02:10 fracpete

```
fixed loading of optional file
```

- gui/explorer/Explorer.html (1.38)

2006-11-17 00:28 fracpete

```
fixed ignoring of randomizing of subsample (subsample was incorrectly generated from original, un-randomized data). thanks to Gabi Schmidberger
```

- gui/visualize/MatrixPanel.html (1.16)

2006-11-16 23:01 fracpete

```
added toOptionSynopsis method for an automated generation of text suitable for the listOptions method
```

- core/Tag.html (1.8)

2006-11-16 22:26 mhall

```
Fixed bug with class priors in the case of a numeric class.
```

- classifiers/rules/DecisionTable.html (1.39)

2006-11-16 20:22 fracpete

```
resampling is now allowed with weight pruning
```

- classifiers/meta/AdaBoostM1.html (1.34)

2006-11-16 20:22 fracpete

```
moved output in setWeights method to debug mode
```

- classifiers/meta/MultiBoostAB.html (1.13)

2006-11-15 20:58 mhall

```
Made the clearLayout method public so that the GUIChooser can call it just before the KF window is closed.
```

- gui/beans/KnowledgeFlowApp.html (1.14)

2006-11-01 03:02 fracpete

```
minimum number of instances is now 1
```

- classifiers/functions/PLSClassifier.html (1.3)

2006-10-31 02:13 fracpete

```
backup object was not properly initialized (pressing "cancel" in the property dialog changes back to an even older object)??? calling setValue a second time fixes that.
```

- gui/PropertyPanel.html (1.12)

2006-10-30 00:01 fracpete

```
incremental loading is no longer unset globally if loader is non-incremental, uses a local variable instead now
```

- gui/SetInstancesPanel.html (1.11)

2006-10-27 03:14 fracpete

```
fixed filters for use in the FilteredClassifier
```

- filters/SimpleFilter.html (1.3)
- filters/supervised/attribute/PLSFilter.html (1.3)
- filters/supervised/instance/Resample.html (1.7)
- filters/supervised/instance/SpreadSubsample.html (1.6)
- filters/supervised/instance/StratifiedRemoveFolds.html (1.5)
- filters/unsupervised/attribute/AbstractTimeSeries.html (1.6)
- filters/unsupervised/attribute/AddCluster.html (1.8)
- filters/unsupervised/attribute/AddID.html (1.5)
- filters/unsupervised/attribute/AddNoise.html (1.6)
- filters/unsupervised/attribute/AddValues.html (1.2)
- filters/unsupervised/attribute/MultiInstanceToPropositional.html (1.6)
- filters/unsupervised/attribute/NumericToNominal.html (1.2)
- filters/unsupervised/attribute/Obfuscate.html (1.5)
- filters/unsupervised/attribute/PropositionalToMultiInstance.html (1.5)
- filters/unsupervised/attribute/RandomProjection.html (1.7)
- filters/unsupervised/attribute/StringToWordVector.html (1.15)
- filters/unsupervised/instance/Randomize.html (1.5)
- filters/unsupervised/instance/RemoveFolds.html (1.4)
- filters/unsupervised/instance/RemoveFrequentValues.html (1.4)
- filters/unsupervised/instance/RemovePercentage.html (1.6)
- filters/unsupervised/instance/RemoveRange.html (1.6)
- filters/unsupervised/instance/Resample.html (1.7)

2006-10-27 03:13 fracpete

```
fixed endless loop in case of multi-instance data in forCapabilities method
added unary class handling in forCapabilities method
```

- core/TestInstances.html (1.6)

2006-10-27 03:12 fracpete

```
capabilities now contain the correct owner, i.e., EM
```

- clusterers/EM.html (1.38)

2006-10-24 19:43 fracpete

```
added "isChanged" method to determine whether the data has been modified by the user
```

- gui/ViewerDialog.html (1.2)

2006-10-18 21:48 fracpete

```
added median for numeric classes
```

- classifiers/meta/Vote.html (1.12)

2006-10-17 22:21 fracpete

```
better exception handling in runClassifier method
```

- classifiers/Classifier.html (1.15)

2006-10-13 00:33 fracpete

```
no longer loses user-provided URL/User/Password/Key columns in KnowledgeFlow (due to calling the reset() method) by defaulting back to values from DatabaseUtils.props
```

- core/converters/DatabaseLoader.html (1.9)

2006-10-04 20:03 fracpete

```
contains Ashraf's bugfixes
indentical instances can now be skipped
```

- core/LinearNN.html (1.4)

2006-10-04 00:54 fracpete

```
uses the new convenience methods from DataSource (setting class index/dataset immediately)
```

- clusterers/ClusterEvaluation.html (1.35)

2006-10-02 02:29 fracpete

```
accidentally, the second element in a bag was tested if there were elements in it. crashed in case there was only one element in the bag. fixed to test the first element.
```

- core/Capabilities.html (1.14)

2006-09-27 00:01 fracpete

```
exception in getStructure() now provides more information on why the data coming from the reader cannot be interpreted as ARFF
```

- core/converters/ArffLoader.html (1.15)

2006-09-25 02:24 fracpete

```
removed final from methods containedBy and hashCode
added serialVersionUID
cleaned up imports
```

- associations/ItemSet.html (1.11)

2006-09-25 01:26 fracpete

```
checks now also the string ID of the underlying tags to determine the selected tag
added toString method
added check of uniqueness of IDs (int and string)
```

- core/SelectedTag.html (1.9)

2006-09-25 01:22 fracpete

```
added GPL preamble and copyright
```

- core/MathematicalExpression.html (1.2)

2006-09-20 07:59 fracpete

```
fixed Javadoc
```

- filters/unsupervised/attribute/InterquartileRange.html (1.2)

2006-09-20 07:58 fracpete

```
fixed Javadoc
```

- filters/unsupervised/attribute/ClusterMembership.html (1.11)

2006-09-20 07:58 fracpete

```
fixed Javadoc
```

- filters/supervised/attribute/ClassOrder.html (1.7)

2006-09-20 07:57 fracpete

```
fixed Javadoc
```

- datagenerators/clusterers/SubspaceClusterDefinition.html (1.3)

2006-09-20 07:57 fracpete

```
fixed Javadoc
```

- classifiers/bayes/net/search/ci/ICSSearchAlgorithm.html (1.6)

2006-09-20 07:56 fracpete

```
fixed Javadoc
cleaned up imports
```

- gui/visualize/PlotData2D.html (1.17)

2006-09-20 07:55 fracpete

```
fixed Javadoc
cleaned up imports
```

- gui/visualize/Plot2D.html (1.23)

2006-09-20 07:54 fracpete

```
fixed Javadoc
cleaned up imports
```

- gui/visualize/ClassPanel.html (1.13)

2006-09-20 07:54 fracpete

```
fixed Javadoc
cleaned up imports
```

- gui/streams/InstanceJoiner.html (1.4)

2006-09-20 07:53 fracpete

```
fixed Javadoc
```

- gui/graphvisualizer/BIFParser.html (1.6)

2006-09-20 07:53 fracpete

```
fixed Javadoc
```

- gui/streams/InstanceEvent.html (1.5)

2006-09-20 07:51 fracpete

```
fixed Javadoc
cleaned up imports
```

- gui/boundaryvisualizer/RemoteBoundaryVisualizerSubTask.html (1.3)

2006-09-20 07:50 fracpete

```
fixed Javadoc
cleaned up imports
```

- gui/boundaryvisualizer/KDDataGenerator.html (1.5)

2006-09-20 07:49 fracpete

```
fixed Javadoc
cleaned up imports
```

- gui/beans/AbstractTrainingSetProducer.html (1.4)

2006-09-20 07:49 fracpete

```
fixed Javadoc
cleaned up imports
```

- gui/beans/BeanConnection.html (1.5)

2006-09-20 07:49 fracpete

```
fixed Javadoc
cleaned up imports
```

- gui/beans/IncrementalClassifierEvent.html (1.7)

2006-09-20 07:48 fracpete

```
fixed Javadoc
```

- gui/ResultHistoryPanel.html (1.23)

2006-09-20 07:48 fracpete

```
fixed Javadoc
```

- gui/HierarchyPropertyParser.html (1.3)

2006-09-20 07:47 fracpete

```
fixed Javadoc
```

- experiment/OutputZipper.html (1.6)

2006-09-20 07:47 fracpete

```
fixed Javadoc
```

- experiment/PairedStatsCorrected.html (1.3)

2006-09-20 07:46 fracpete

```
fixed Javadoc
```

- estimators/NNConditionalEstimator.html (1.6)

2006-09-20 07:46 fracpete

```
fixed Javadoc
```

- estimators/NDConditionalEstimator.html (1.5)

2006-09-20 07:46 fracpete

```
fixed Javadoc
```

- estimators/MahalanobisEstimator.html (1.6)

2006-09-20 07:45 fracpete

```
fixed Javadoc
```

- estimators/KKConditionalEstimator.html (1.6)

2006-09-20 07:44 fracpete

```
fixed Javadoc
```

- estimators/KDConditionalEstimator.html (1.6)

2006-09-20 07:44 fracpete

```
fixed Javadoc
```

- estimators/EstimatorUtils.html (1.2)

2006-09-20 07:44 fracpete

```
fixed Javadoc
```

- estimators/Estimator.html (1.7)

2006-09-20 07:43 fracpete

```
fixed Javadoc
```

- estimators/DNConditionalEstimator.html (1.6)

2006-09-20 07:42 fracpete

```
fixed Javadoc
```

- estimators/DDConditionalEstimator.html (1.6)

2006-09-20 07:42 fracpete

```
fixed Javadoc
```

- estimators/DKConditionalEstimator.html (1.6)

2006-09-20 07:42 fracpete

```
fixed Javadoc
```

- estimators/CheckEstimator.html (1.2)

2006-09-20 07:41 fracpete

```
fixed Javadoc
```

- core/xml/XMLDocument.html (1.6)

2006-09-20 07:41 fracpete

```
fixed Javadoc
```

- core/xml/XMLSerialization.html (1.15)

2006-09-20 07:40 fracpete

```
fixed Javadoc
```

- core/matrix/SingularValueDecomposition.html (1.2)

2006-09-20 07:40 fracpete

```
fixed Javadoc
```

- core/matrix/QRDecomposition.html (1.2)

2006-09-20 07:39 fracpete

```
fixed Javadoc
```

- core/matrix/LUDecomposition.html (1.2)

2006-09-20 07:39 fracpete

```
fixed Javadoc
```

- core/matrix/LinearRegression.html (1.3)

2006-09-20 07:39 fracpete

```
fixed Javadoc
```

- core/matrix/Matrix.html (1.6)

2006-09-20 07:38 fracpete

```
fixed Javadoc
```

- core/matrix/IntVector.html (1.2)

2006-09-20 07:38 fracpete

```
fixed Javadoc
```

- core/matrix/EigenvalueDecomposition.html (1.2)

2006-09-20 07:37 fracpete

```
fixed Javadoc
```

- core/matrix/CholeskyDecomposition.html (1.2)

2006-09-20 07:37 fracpete

```
fixed Javadoc
```

- core/matrix/DoubleVector.html (1.2)

2006-09-20 07:36 fracpete

```
fixed Javadoc
```

- core/converters/Loader.html (1.8)

2006-09-20 07:35 fracpete

```
fixed Javadoc
```

- core/SerializedObject.html (1.8)

2006-09-20 07:35 fracpete

```
fixed Javadoc
```

- core/Statistics.html (1.9)

2006-09-20 07:34 fracpete

```
fixed Javadoc
```

- core/ProtectedProperties.html (1.3)

2006-09-20 07:34 fracpete

```
fixed Javadoc
```

- core/RandomVariates.html (1.2)

2006-09-20 07:33 fracpete

```
fixed Javadoc
```

- core/NearestNeighbourSearch.html (1.3)

2006-09-20 07:32 fracpete

```
fixed Javadoc
```

- core/Memory.html (1.5)

2006-09-20 07:31 fracpete

```
fixed Javadoc
```

- core/Instances.html (1.66)

2006-09-20 07:30 fracpete

```
fixed Javadoc
```

- core/FastVector.html (1.12)

2006-09-20 07:30 fracpete

```
fixed Javadoc
```

- core/EuclideanDistance.html (1.8)

2006-09-20 07:29 fracpete

```
fixed Javadoc
```

- core/Debug.html (1.5)

2006-09-20 07:29 fracpete

```
fixed Javadoc
```

- core/ContingencyTables.html (1.6)

2006-09-20 07:28 fracpete

```
fixed Javadoc
```

- core/AlgVector.html (1.6)

2006-09-20 07:28 fracpete

```
fixed Javadoc
```

- clusterers/XMeans.html (1.19)

2006-09-20 07:27 fracpete

```
fixed Javadoc
```

- clusterers/Clusterer.html (1.14)

2006-09-20 05:45 fracpete

```
fixed Javadoc
```

- classifiers/trees/m5/Rule.html (1.12)

2006-09-20 05:44 fracpete

```
fixed Javadoc
```

- classifiers/trees/m5/M5Base.html (1.16)

2006-09-20 05:44 fracpete

```
fixed Javadoc
```

- classifiers/trees/lmt/LMTNode.html (1.5)

2006-09-20 05:43 fracpete

```
fixed Javadoc
```

- classifiers/trees/j48/NBTreeClassifierTree.html (1.3)

2006-09-20 05:43 fracpete

```
fixed Javadoc
```

- classifiers/trees/j48/BinC45Split.html (1.11)

2006-09-20 05:43 fracpete

```
fixed Javadoc
```

- classifiers/trees/j48/InfoGainSplitCrit.html (1.7)

2006-09-20 05:42 fracpete

```
fixed Javadoc
```

- classifiers/trees/adtree/TwoWayNumericSplit.html (1.3)

2006-09-20 05:41 fracpete

```
fixed Javadoc
```

- classifiers/trees/adtree/TwoWayNominalSplit.html (1.3)

2006-09-20 05:41 fracpete

```
fixed Javadoc
```

- classifiers/trees/UserClassifier.html (1.26)

2006-09-20 05:40 fracpete

```
fixed Javadoc
```

- classifiers/trees/RandomTree.html (1.16)

2006-09-20 05:39 fracpete

```
fixed Javadoc
```

- classifiers/rules/part/PruneableDecList.html (1.7)

2006-09-20 05:37 fracpete

```
fixed Javadoc
```

- classifiers/rules/part/ClassifierDecList.html (1.10)

2006-09-20 05:37 fracpete

```
fixed Javadoc
```

- classifiers/rules/Rule.html (1.5)

2006-09-20 05:36 fracpete

```
fixed Javadoc
```

- classifiers/rules/Ridor.html (1.17)

2006-09-20 05:35 fracpete

```
fixed Javadoc
```

- classifiers/misc/FLR.html (1.9)

2006-09-20 05:35 fracpete

```
fixed Javadoc
```

- classifiers/rules/JRip.html (1.19)

2006-09-20 05:34 fracpete

```
fixed Javadoc
```

- classifiers/mi/TLDSimple.html (1.4)

2006-09-20 05:34 fracpete

```
fixed Javadoc
```

- classifiers/mi/TLD.html (1.4)

2006-09-20 05:33 fracpete

```
fixed Javadoc
```

- classifiers/mi/MINND.html (1.4)

2006-09-20 05:33 fracpete

```
fixed Javadoc
```

- classifiers/mi/CitationKNN.html (1.5)

2006-09-20 05:32 fracpete

```
fixed Javadoc
```

- classifiers/functions/pace/PaceMatrix.html (1.4)

2006-09-20 05:30 fracpete

```
fixed Javadoc
```

- classifiers/functions/SMO.html (1.63)

2006-09-20 05:29 fracpete

```
fixed Javadoc
```

- classifiers/functions/MultilayerPerceptron.html (1.7)

2006-09-20 05:29 fracpete

```
fixed Javadoc
```

- classifiers/functions/LeastMedSq.html (1.15)

2006-09-20 05:29 fracpete

```
fixed Javadoc
```

- classifiers/bayes/net/BayesNetGenerator.html (1.11)

2006-09-20 05:28 fracpete

```
fixed Javadoc
```

- classifiers/CostMatrix.html (1.13)

2006-09-20 05:28 fracpete

```
fixed Javadoc
```

- classifiers/bayes/ComplementNaiveBayes.html (1.7)

2006-09-20 05:27 fracpete

```
uses now ConverterUtils.DataSource instead of a BufferedReader for just  ARFF files
```

- associations/AssociatorEvaluation.html (1.3)

2006-09-20 05:26 fracpete

```
fixed Javadoc
```

- associations/Tertius.html (1.8)

2006-09-19 05:06 fracpete

```
removed dead code: preprocess(Instance)
```

- filters/unsupervised/attribute/Wavelet.html (1.2)

2006-09-19 04:59 fracpete

```
moved to ConverterUtils to determine loader, instead of only ARFF files
class index is only set if loader returned dataset without one (new XML format now enables arbitrary class attribute definition)
```

- experiment/Experiment.html (1.25)

2006-09-19 04:55 fracpete

```
displays now all the extensions that the specific loader can handle
```

- gui/beans/LoaderCustomizer.html (1.12)

2006-09-19 04:54 fracpete

```
fixed handling of Loaders and Savers
```

- gui/beans/xml/XMLBeans.html (1.7)

2006-09-19 04:53 fracpete

```
JFileChooser got changed to ConverterFileChooser
saving to a file respects the Capabilities of the data, ie only Converters that can handle that kind of data will be displayed
```

- gui/arffviewer/ArffViewerMainPanel.html (1.5)

2006-09-19 04:53 fracpete

```
superceded by weka.gui.ConverterFileChooser
```

- gui/arffviewer/FileChooser.html (1.4)

2006-09-19 04:49 fracpete

```
added getExtensions() method to return stored extensions
```

- gui/ExtensionFileFilter.html (1.6)

2006-09-19 04:46 fracpete

```
added Capabilities generation
main method now uses runFileSaver
no longer IncrementalConverter
```

- core/converters/SerializedInstancesSaver.html (1.5)

2006-09-19 04:45 fracpete

```
added getFileExtensions()
removed redundant code due to AbstractFileLoader
main method now uses runFileLoader
```

- core/converters/SerializedInstancesLoader.html (1.11)

2006-09-19 04:45 fracpete

```
added Capabilities generation
main method now uses runFileSaver
fixed option handling (changed -C to -c to make it consistent with other savers)
```

- core/converters/LibSVMSaver.html (1.2)

2006-09-19 04:41 fracpete

```
added getFileExtensions()
removed redundant code due to AbstractFileLoader
main method now uses runFileLoader
```

- core/converters/LibSVMLoader.html (1.2)

2006-09-19 04:39 fracpete

```
added Capabilities generation
main method now uses runFileSaver
```

- core/converters/CSVSaver.html (1.5)

2006-09-19 04:39 fracpete

```
added getFileExtensions()
removed redundant code due to AbstractFileLoader
main method now uses runFileLoader
```

- core/converters/CSVLoader.html (1.11)

2006-09-19 04:37 fracpete

```
added Capabilities generation
removed obsolete setDestination calls
listOptions now uses the ones from its ancestor
fixed setting of class attribute in setOptions
main method now uses runFileSaver
```

- core/converters/C45Saver.html (1.5)

2006-09-19 04:36 fracpete

```
added getFileExtensions()
removed redundant code due to AbstractFileLoader
main method now uses runFileLoader
```

- core/converters/C45Loader.html (1.12)

2006-09-19 04:32 fracpete

```
added Capabilities generation
main method now uses runFileSaver
```

- core/converters/ArffSaver.html (1.5)

2006-09-19 04:30 fracpete

```
Savers are now CapabilitiesHandlers
```

- core/converters/AbstractSaver.html (1.3)

2006-09-19 04:29 fracpete

```
m_writer now transient (wasn't serializable)
setFile now automatically performs setDestination
added makeOptionStr and runFileSaver to remove duplicate code in savers
```

- core/converters/AbstractFileSaver.html (1.3)

2006-09-19 04:27 fracpete

```
added getFileExtensions() to return array of extensions (e.g., for C45 and XML loader/saver)
```

- core/converters/FileSourcedConverter.html (1.3)

2006-09-19 04:21 fracpete

```
cleaned up code
fixed Javadoc
```

- core/Version.html (1.6)

2006-09-19 04:20 fracpete

```
added methods to work on classnames instead of classes
fixed Javadoc
```

- core/ClassDiscovery.html (1.4)

2006-09-19 04:17 fracpete

```
abstract super class for file loaders
```

- core/converters/AbstractFileLoader.html (1.1)

2006-09-18 21:20 fracpete

```
fixed listOptions/Javadoc
```

- attributeSelection/FCBFSearch.html (1.6)

2006-09-07 22:58 fracpete

```
variable processed_InstanceID wasn't initialized in buildClusterer - fixed
```

- clusterers/DBScan.html (1.7)

2006-09-06 04:57 fracpete

```
added distributionForInstance method/probability estimates according to Yasser EL-Manzalawy's original code
rewrote transfer for Weka data into libsvm's sparse data format (more efficient now)
Capabilities are now based on the SVMType that has been selected
```

- classifiers/functions/LibSVM.html (1.7)

2006-09-05 21:39 fracpete

```
splash screen for the new GUI interface
```

- gui/images/weka\_splash.gif (1.1)

2006-09-05 05:08 fracpete

```
background for new main GUI
```

- gui/images/weka\_background.gif (1.1)

2006-09-05 05:06 fracpete

```
SimpleCLI split into panel and frame that uses that panel (necessary for integration in new main GUI)
```

- gui/SimpleCLI.html (1.9)

2006-09-05 05:05 fracpete

```
a little helper class for browser related stuff
```

- gui/BrowserHelper.html (1.1)

2006-09-04 22:03 fracpete

```
year is ommitted now if missing (suppresses those ugly empty parentheses)
```

- core/TechnicalInformation.html (1.7)

2006-09-04 05:35 fracpete

```
gaussian noise (m_delta) now back to 1.0 as default value like in initial import (Javadoc disagreed back then)
```

- classifiers/functions/GaussianProcesses.html (1.7)

2006-09-04 04:22 mhall

```
Modified so that the user can choose to have the class attribute unset,
```

- gui/beans/ClassAssigner.html (1.11)

2006-09-04 04:21 mhall

```
Modified so that "no class" is an option from the drop-down box (and 0 can be used from the text field to indicate the same thing).
```

- gui/beans/ClassAssignerCustomizer.html (1.5)

2006-09-03 23:19 fracpete

```
fixed getOptions (-Q was inverted)
```

- classifiers/bayes/net/search/global/GlobalScoreSearchAlgorithm.html (1.8)

2006-08-28 05:38 fracpete

```
removed obsolete code, i.e., left-overs from CheckClassifier (number of test instances)
fixed Javadoc
```

- clusterers/CheckClusterer.html (1.7)

2006-08-28 05:37 fracpete

```
modified determination of position of string attribute
```

- classifiers/functions/supportVector/StringKernel.html (1.5)

2006-08-28 05:36 fracpete

```
added makeCopies method (only for convencience in CheckKernel class)
```

- classifiers/functions/supportVector/Kernel.html (1.5)

2006-08-28 05:35 fracpete

```
for testing kernels
```

- classifiers/functions/supportVector/CheckKernel.html (1.1)

2006-08-28 05:35 fracpete

```
used for comparing kernels
```

- classifiers/functions/supportVector/KernelEvaluation.html (1.1)

2006-08-28 05:32 fracpete

```
removed obsolete code, i.e., left-overs from CheckClassifier (number of test instances)
updated Javadoc
```

- attributeSelection/CheckAttributeSelection.html (1.4)

2006-08-28 05:31 fracpete

```
removed obsolete code, i.e., left-overs from CheckClassifier (number of test instances)
```

- associations/CheckAssociator.html (1.3)

2006-08-28 05:00 fracpete

```
cleaned up imports
fixed Javadoc ("all" is not  supported)
added serialVersionUID
```

- core/Range.html (1.16)

2006-08-28 05:00 fracpete

```
cleaned up imports
fixed Javadoc
added serialVersionUID
```

- core/SingleIndex.html (1.6)

2006-08-28 04:59 fracpete

```
added method attributeTypeToString to turn an attribute into a string, e.g., for regression tests or exceptions
```

- core/CheckScheme.html (1.3)

2006-08-24 22:26 fracpete

```
added tiptext methods
```

- classifiers/trees/M5P.html (1.8)

2006-08-24 21:44 fracpete

```
introduced -1 as cache size for turning the cache off
fixed initVars - kernels weren't initialized correctly for cache_size=0 and being loaded in the GOE from a serialized, trained model (generated complete crap numbers with GaussianProcesses!)
```

- classifiers/functions/supportVector/CachedKernel.html (1.6)

2006-08-24 02:12 fracpete

```
moved into "attribute" package
```

- filters/unsupervised/instance/InterquartileRange.html (1.2)

2006-08-23 02:17 fracpete

```
new classifier, submitted by Liangxiao Jiang
upgraded it to latest developer CVS
added -I parameter to output the mutual-information data in the toString method (toString was missing anyway)
```

- classifiers/bayes/WAODE.html (1.1)

2006-08-22 23:31 fracpete

```
setMinimumNumberInstances is explicitly set to 0, otherwise the testInputFormat method will fail
```

- filters/unsupervised/instance/RemoveMisclassified.html (1.6)

2006-08-22 23:30 fracpete

```
setMinimumNumberInstances is explicitly set to 0, otherwise the testInputFormat method will fail
```

- filters/supervised/attribute/AttributeSelection.html (1.6)

2006-08-22 23:28 fracpete

```
fixed typos in Javadoc (referred to classifier instead of filter)
```

- filters/MultiFilter.html (1.6)

2006-08-22 03:41 fracpete

```
updated Javadoc (PotentialClassIgnorer is now OptionHandler) and option handling
```

- filters/unsupervised/attribute/PKIDiscretize.html (1.7)

2006-08-22 03:34 fracpete

```
updated Javadoc (PotentialClassIgnorer is now OptionHandler) and option handling
```

- filters/unsupervised/attribute/Discretize.html (1.11)
- filters/unsupervised/attribute/MathExpression.html (1.7)

2006-08-22 03:34 fracpete

```
updated Javadoc (PotentialClassIgnorer is now OptionHandler)
```

- filters/unsupervised/attribute/NumericToBinary.html (1.6)

2006-08-22 03:33 fracpete

```
updated Javadoc (PotentialClassIgnorer is now OptionHandler)
```

- filters/unsupervised/attribute/Center.html (1.2)
- filters/unsupervised/attribute/Normalize.html (1.7)
- filters/unsupervised/attribute/ReplaceMissingValues.html (1.7)
- filters/unsupervised/attribute/Standardize.html (1.7)

2006-08-22 03:32 fracpete

```
added get method for m_IgnoreClass
implements now OptionHandler ("-unset-class-temporarily")
```

- filters/unsupervised/attribute/PotentialClassIgnorer.html (1.4)

2006-08-22 03:30 fracpete

```
filter for setting and unsetting the class index
```

- filters/unsupervised/attribute/ClassAssigner.html (1.1)

2006-08-17 04:52 fracpete

```
fixed Javadoc
```

- core/Tee.html (1.3)

2006-08-17 04:03 uid62904

```
now derived from RandomizableClusterer
```

- clusterers/SimpleKMeans.html (1.27)

2006-08-17 04:02 fracpete

```
now derived from RandomizableClusterer
```

- clusterers/FarthestFirst.html (1.7)

2006-08-17 03:59 fracpete

```
now derived from RandomizableClusterer
made an incremental clusterer
```

- clusterers/Cobweb.html (1.21)

2006-08-17 03:58 fracpete

```
added support for incremental clusterers
```

- clusterers/UpdateableClusterer.html (1.1)

2006-08-17 03:57 fracpete

```
additional abstract superclasses to avoid redundant code in derived classes
```

- clusterers/RandomizableClusterer.html (1.1)
- clusterers/RandomizableDensityBasedClusterer.html (1.1)
- clusterers/RandomizableSingleClustererEnhancer.html (1.1)

2006-08-17 03:44 fracpete

```
for default values in the Explorer
```

- gui/explorer/ExplorerDefaults.html (1.1)

2006-08-17 03:42 fracpete

```
added main method
added serialVersionUID
fixed Javadoc
```

- gui/experiment/ExperimenterDefaults.html (1.4)

2006-08-17 03:41 fracpete

```
Capabilities now based on the base classifier
```

- attributeSelection/WrapperSubsetEval.html (1.28)

2006-08-17 03:41 fracpete

```
fixed exception text in search method that tests no longer fail
```

- attributeSelection/RaceSearch.html (1.20)

2006-08-17 03:40 fracpete

```
Capabilities now based on the base classifier
```

- attributeSelection/ClassifierSubsetEval.html (1.16)

2006-08-16 03:50 gs23

```
its interface Estimator changed from interface to abstract class,  added capabilities functions
```

- classifiers/bayes/net/estimate/DiscreteEstimatorBayes.html (1.5)

2006-08-16 03:27 gs23

```
interface to set type of estimator to incremental
```

- estimators/IncrementalEstimator.html (1.1)

2006-08-16 03:12 gs23

```
its interface Estimator changed from interface to abstract class,  added capabilities functions
```

- estimators/NormalEstimator.html (1.6)

2006-08-16 03:12 gs23

```
its interface Estimator changed from interface to abstract class,  added capabilities functions
```

- estimators/PoissonEstimator.html (1.5)

2006-08-16 03:10 gs23

```
its interface Estimator changed from interface to abstract class,  added capabilities functions
```

- estimators/KernelEstimator.html (1.6)

2006-08-16 03:09 gs23

```
its interface Estimator changed from interface to abstract class,  added capabilities functions
```

- estimators/DiscreteEstimator.html (1.8)

2006-08-16 02:56 gs23

```
organized imports
```

- estimators/ConditionalEstimator.html (1.5)

2006-08-11 00:37 fracpete

```
moved flushInput() after the data has been processed, in case determineOutputFormat needs to access the input data
```

- filters/SimpleBatchFilter.html (1.6)

2006-08-10 06:41 fracpete

```
setOptions now defaults to CfsSubsetEval/BestFirst if no Evaluator/Search provided instead of throwing an Exception
fixed Javadoc
```

- classifiers/meta/AttributeSelectedClassifier.html (1.24)

2006-08-10 06:40 fracpete

```
fixed getOptions and setOptions
```

- classifiers/lazy/LWL.html (1.18)

2006-08-10 06:36 fracpete

```
fixed setOptions
```

- associations/PredictiveApriori.html (1.9)

2006-08-10 06:34 fracpete

```
now based on weka.core.Check
```

- core/CheckOptionHandler.html (1.10)

2006-08-10 06:33 fracpete

```
abstract superclass for CheckScheme and CheckOptionHandler
```

- core/Check.html (1.1)

2006-08-10 03:32 fracpete

```
incorporated Remco's fix regarding nearly-endless-loop in the RegSMOImproved (happened with some weighted datasets)
```

- classifiers/functions/supportVector/RegSMO.html (1.2)

2006-08-10 02:48 fracpete

```
added main method to list all installed themes
since setting the system default theme can crash Weka under Linux/Gnome we don't set the system theme, if we're on a Linux box (i.e., system property "os.name" == "linux")
```

- gui/LookAndFeel.html (1.2)

2006-08-09 22:50 fracpete

```
setOptions no longer throws an Exception if clusterer is not given, defaults to SimpleKMeans now
```

- clusterers/MakeDensityBasedClusterer.html (1.10)

2006-08-09 21:47 fracpete

```
added getCapabilities method to code examples
```

- filters/SimpleStreamFilter.html (1.6)

2006-08-09 06:36 fracpete

```
main method now uses runClassifier
```

- classifiers/trees/ADTree.html (1.6)
- classifiers/trees/DecisionStump.html (1.21)
- classifiers/trees/Id3.html (1.19)
- classifiers/trees/J48.html (1.5)
- classifiers/trees/LMT.html (1.9)
- classifiers/trees/NBTree.html (1.8)
- classifiers/trees/REPTree.html (1.24)

2006-08-09 06:36 fracpete

```
main method now uses runClassifier
```

- classifiers/trees/RandomForest.html (1.11)

2006-08-09 06:34 fracpete

```
main method now uses runClassifier
```

- classifiers/rules/ConjunctiveRule.html (1.13)
- classifiers/rules/M5Rules.html (1.9)
- classifiers/rules/NNge.html (1.6)
- classifiers/rules/PART.html (1.7)
- classifiers/rules/Prism.html (1.21)
- classifiers/rules/ZeroR.html (1.14)

2006-08-09 06:33 fracpete

```
main method now uses runClassifier
throws a WekaException now if no rule could be found instead of crashing with a NullPointerException in classifyInstance
```

- classifiers/rules/OneR.html (1.21)

2006-08-09 06:30 fracpete

```
main method now uses runClassifier
```

- classifiers/mi/MDD.html (1.3)
- classifiers/mi/MIDD.html (1.3)
- classifiers/mi/MIEMDD.html (1.5)
- classifiers/mi/MILR.html (1.3)
- classifiers/mi/MIOptimalBall.html (1.4)
- classifiers/mi/MISMO.html (1.4)
- classifiers/mi/MISVM.html (1.4)

2006-08-09 06:30 fracpete

```
main method now uses runClassifier
```

- classifiers/misc/HyperPipes.html (1.18)
- classifiers/misc/VFI.html (1.13)

2006-08-09 06:27 fracpete

```
main method now uses runClassifier
```

- classifiers/meta/AdditiveRegression.html (1.21)
- classifiers/meta/Bagging.html (1.37)
- classifiers/meta/CVParameterSelection.html (1.33)
- classifiers/meta/ClassificationViaRegression.html (1.25)
- classifiers/meta/CostSensitiveClassifier.html (1.26)
- classifiers/meta/Dagging.html (1.5)
- classifiers/meta/Decorate.html (1.8)
- classifiers/meta/END.html (1.6)
- classifiers/meta/Grading.html (1.11)
- classifiers/meta/LogitBoost.html (1.37)
- classifiers/meta/MetaCost.html (1.22)
- classifiers/meta/MultiClassClassifier.html (1.42)
- classifiers/meta/MultiScheme.html (1.22)
- classifiers/meta/OrdinalClassClassifier.html (1.16)
- classifiers/meta/RacedIncrementalLogitBoost.html (1.10)

2006-08-09 06:27 fracpete

```
main method now uses runClassifier
```

- classifiers/meta/RandomCommittee.html (1.10)
- classifiers/meta/RegressionByDiscretization.html (1.33)
- classifiers/meta/Stacking.html (1.30)
- classifiers/meta/StackingC.html (1.12)
- classifiers/meta/ThresholdSelector.html (1.37)

2006-08-09 06:24 fracpete

```
updated main method (can't use runClassifier method unfortunately)
```

- classifiers/meta/EnsembleSelection.html (1.2)

2006-08-09 06:22 fracpete

```
main method now uses runClassifier
getCapabilities now returns the Capabilities of the filter
```

- classifiers/meta/FilteredClassifier.html (1.25)

2006-08-09 06:20 fracpete

```
added the dependencies
```

- clusterers/FilteredClusterer.html (1.2)

2006-08-09 06:20 fracpete

```
added the dependencies
```

- clusterers/SingleClustererEnhancer.html (1.2)

2006-08-09 05:41 fracpete

```
main method now uses runClassifier
```

- classifiers/meta/nestedDichotomies/ClassBalancedND.html (1.6)
- classifiers/meta/nestedDichotomies/DataNearBalancedND.html (1.6)
- classifiers/meta/nestedDichotomies/ND.html (1.6)

2006-08-09 05:40 fracpete

```
main method now uses runClassifier
```

- classifiers/lazy/IB1.html (1.18)
- classifiers/lazy/IBk.html (1.38)
- classifiers/lazy/KStar.html (1.7)
- classifiers/lazy/LBR.html (1.10)

2006-08-09 05:38 fracpete

```
main method now uses runClassifier
```

- classifiers/functions/LinearRegression.html (1.22)
- classifiers/functions/Logistic.html (1.36)
- classifiers/functions/PaceRegression.html (1.7)
- classifiers/functions/RBFNetwork.html (1.7)
- classifiers/functions/SMOreg.html (1.12)
- classifiers/functions/SVMreg.html (1.2)
- classifiers/functions/SimpleLinearRegression.html (1.8)
- classifiers/functions/SimpleLogistic.html (1.11)
- classifiers/functions/VotedPerceptron.html (1.21)
- classifiers/functions/Winnow.html (1.12)

2006-08-09 05:34 fracpete

```
main method now uses runClassifier
```

- classifiers/bayes/AODE.html (1.16)
- classifiers/bayes/BayesNet.html (1.29)
- classifiers/bayes/HNB.html (1.8)
- classifiers/bayes/NaiveBayes.html (1.20)
- classifiers/bayes/NaiveBayesMultinomial.html (1.13)
- classifiers/bayes/NaiveBayesSimple.html (1.18)
- classifiers/bayes/NaiveBayesUpdateable.html (1.8)

2006-08-09 05:15 fracpete

```
added Capabilities support
main method now uses runFilter
```

- filters/unsupervised/instance/NonSparseToSparse.html (1.3)
- filters/unsupervised/instance/Normalize.html (1.5)
- filters/unsupervised/instance/RemoveWithValues.html (1.11)
- filters/unsupervised/instance/SparseToNonSparse.html (1.4)

2006-08-09 05:11 fracpete

```
added Capabilities support
main method now uses runFilter
```

- filters/unsupervised/attribute/Add.html (1.5)
- filters/unsupervised/attribute/ChangeDateFormat.html (1.5)
- filters/unsupervised/attribute/Copy.html (1.5)
- filters/unsupervised/attribute/FirstOrder.html (1.7)
- filters/unsupervised/attribute/MakeIndicator.html (1.4)

2006-08-09 05:11 fracpete

```
added Capabilities support
main method now uses runFilter
```

- filters/unsupervised/attribute/MergeTwoValues.html (1.7)
- filters/unsupervised/attribute/NominalToBinary.html (1.10)
- filters/unsupervised/attribute/NumericTransform.html (1.7)
- filters/unsupervised/attribute/Remove.html (1.7)
- filters/unsupervised/attribute/RemoveType.html (1.6)
- filters/unsupervised/attribute/RemoveUseless.html (1.7)
- filters/unsupervised/attribute/Reorder.html (1.4)
- filters/unsupervised/attribute/StringToNominal.html (1.7)
- filters/unsupervised/attribute/SwapValues.html (1.7)
- filters/unsupervised/attribute/TimeSeriesDelta.html (1.5)
- filters/unsupervised/attribute/TimeSeriesTranslate.html (1.6)

2006-08-09 05:02 fracpete

```
added Capabilities support
main method now uses runFilter
setOptions no longer throws an Exception if expression is not given, defaults to " a1^2" now
added globalInfo method
```

- filters/unsupervised/attribute/AddExpression.html (1.6)

2006-08-09 04:58 fracpete

```
added Capabilities support
main method now uses runFilter
```

- filters/supervised/attribute/Discretize.html (1.6)
- filters/supervised/attribute/NominalToBinary.html (1.6)

2006-08-09 04:50 fracpete

```
added Capabilities support
main method now uses runFilter
added serialVersionUID
```

- filters/AllFilter.html (1.9)

2006-08-09 04:38 fracpete

```
main method now uses runDataGenerator
```

- datagenerators/clusterers/BIRCHCluster.html (1.6)
- datagenerators/clusterers/SubspaceCluster.html (1.3)

2006-08-09 04:37 fracpete

```
main method now uses runDataGenerator
```

- datagenerators/classifiers/classification/Agrawal.html (1.5)
- datagenerators/classifiers/classification/BayesNet.html (1.3)
- datagenerators/classifiers/classification/LED24.html (1.4)
- datagenerators/classifiers/classification/RDG1.html (1.3)
- datagenerators/classifiers/classification/RandomRBF.html (1.3)

2006-08-09 04:37 fracpete

```
main method now uses runDataGenerator
```

- datagenerators/classifiers/regression/Expression.html (1.4)
- datagenerators/classifiers/regression/MexicanHat.html (1.3)

2006-08-09 04:36 fracpete

```
added static method runDataGenerator to avoid redundant code in the main method in derived classes
```

- datagenerators/DataGenerator.html (1.4)

2006-08-09 04:13 fracpete

```
added Capabilities support
main method now uses runEvaluator
```

- attributeSelection/SVMAttributeEval.html (1.22)

2006-08-09 04:13 fracpete

```
added Capabilities support
main method now uses runEvaluator
```

- attributeSelection/SymmetricalUncertAttributeEval.html (1.18)
- attributeSelection/SymmetricalUncertAttributeSetEval.html (1.5)

2006-08-09 04:12 fracpete

```
added Capabilities support
main method now uses runEvaluator
fixed getOptions method
```

- attributeSelection/ReliefFAttributeEval.html (1.19)

2006-08-09 04:11 fracpete

```
setOptions no longer throws an Exception if attribute evaluator is not given (defaults to GainRatioAttributeEval now)
```

- attributeSelection/RankSearch.html (1.15)

2006-08-09 04:09 fracpete

```
fixed getOptions and getSearchPercent
```

- attributeSelection/RandomSearch.html (1.16)

2006-08-09 04:07 fracpete

```
added Capabilities support
main method now uses runEvaluator
fixed modifying of data in the buildEvaluator method
```

- attributeSelection/PrincipalComponents.html (1.33)

2006-08-09 04:05 fracpete

```
added Capabilities support
main method now uses runEvaluator
```

- attributeSelection/OneRAttributeEval.html (1.17)

2006-08-09 04:05 fracpete

```
added Capabilities support
main method now uses runEvaluator
```

- attributeSelection/InfoGainAttributeEval.html (1.17)

2006-08-09 04:04 fracpete

```
fixed ArrayOutOfBounds exception in method "select" if no good member was found. now returns the first one in that case.
```

- attributeSelection/GeneticSearch.html (1.16)

2006-08-09 04:02 fracpete

```
added Capabilities support
main method now uses runEvaluator
```

- attributeSelection/GainRatioAttributeEval.html (1.19)

2006-08-09 04:00 fracpete

```
added Capabilities support
main method now uses runEvaluator
fixed modifying of data in the buildEvaluator method
```

- attributeSelection/ConsistencySubsetEval.html (1.13)

2006-08-09 04:00 fracpete

```
m_evaluations is now reset every time the method "search" is called
```

- attributeSelection/ExhaustiveSearch.html (1.13)

2006-08-09 03:57 fracpete

```
added Capabilities support
main method now uses runEvaluator
```

- attributeSelection/ChiSquaredAttributeEval.html (1.11)

2006-08-09 03:56 fracpete

```
added Capabilities support
main method now uses runEvaluator
```

- attributeSelection/CfsSubsetEval.html (1.23)

2006-08-09 03:54 fracpete

```
added makeCopies method
```

- attributeSelection/ASSearch.html (1.9)

2006-08-09 03:54 fracpete

```
added Capabilities support
added static method runEvaluator to eliminate redundant code in the main methods
```

- attributeSelection/ASEvaluation.html (1.11)

2006-08-09 03:42 fracpete

```
main method now uses runClusterer
```

- clusterers/OPTICS.html (1.6)

2006-08-09 03:29 fracpete

```
main method now uses runJavadoc
```

- core/GlobalInfoJavadoc.html (1.5)
- core/OptionHandlerJavadoc.html (1.6)
- core/TechnicalInformationHandlerJavadoc.html (1.4)

2006-08-09 03:28 fracpete

```
main method now uses runJavadoc
added setting  of "-silent" parameter for all Javadoc generators
```

- core/AllJavadoc.html (1.3)

2006-08-09 03:27 fracpete

```
added "-silent" parameter to suppress output in the console
added static runJavadoc method to eliminate redundant code in the main methods of derived classes
```

- core/Javadoc.html (1.5)

2006-08-09 03:25 fracpete

```
fixed Exception in case an attribute is a relational one and has a missing value
```

- core/RelationalLocator.html (1.2)
- core/StringLocator.html (1.3)

2006-08-09 03:19 fracpete

```
main method now uses runAssociator
added getAllTheRules method to access the generated rules
```

- associations/Apriori.html (1.26)

2006-08-09 03:17 fracpete

```
added methods makeCopy and makeCopies
added static method runAssociator for unified commandline calls (removes redundant code from the main methods)
```

- associations/Associator.html (1.7)

2006-08-03 05:04 fracpete

```
Loader based on the TextDirectoryToArff tool to import directories of text files into an Instances object
```

- core/converters/TextDirectoryLoader.html (1.1)

2006-07-31 03:12 fracpete

```
Capabilities tests can now be turned on/off via the weka/core/Capabilities.props file (useful for experiments with nested classifiers/filters and large datasets)
```

- core/Capabilities.props (1.1)

2006-07-24 04:02 fracpete

```
now derived from weka.experiment.DatabaseUtils to avoid duplicate code
```

- core/converters/DatabaseConnection.html (1.5)

2006-07-02 02:07 fracpete

```
cleaned up imports
added right-click menu for history (based on code from weka.gui.explorer.ClassifierPanel)
```

- gui/beans/TextViewer.html (1.9)

2006-06-30 05:33 fracpete

```
branches:  1.2.2;
added comments for supported mappings
```

- experiment/DatabaseUtils.props.mssqlserver (1.2)
- experiment/DatabaseUtils.props.mysql (1.2)
- experiment/DatabaseUtils.props.oracle (1.2)
- experiment/DatabaseUtils.props.postgresql (1.2)

2006-06-30 05:33 fracpete

```
added comments for supported mappings
```

- experiment/DatabaseUtils.props.hsql (1.3)

2006-06-30 05:33 fracpete

```
branches:  1.3.2;
added comments for supported mappings
```

- experiment/DatabaseUtils.props.odbc (1.3)

2006-06-30 01:55 fracpete

```
EnsembleSelection contribution by Robert Jung (mrbobjung at gmail dot com) and David Michael (dam68 at cornell dot edu)
```

- gui/ensembleLibraryEditor/tree/CheckBoxNode.html (1.1)
- gui/ensembleLibraryEditor/tree/CheckBoxNodeEditor.html (1.1)
- gui/ensembleLibraryEditor/tree/DefaultNode.html (1.1)
- gui/ensembleLibraryEditor/tree/GenericObjectNode.html (1.1)
- gui/ensembleLibraryEditor/tree/GenericObjectNodeEditor.html (1.1)
- gui/ensembleLibraryEditor/tree/InvalidInputException.html (1.1)
- gui/ensembleLibraryEditor/tree/ModelTreeNodeEditor.html (1.1)
- gui/ensembleLibraryEditor/tree/ModelTreeNodeRenderer.html (1.1)
- gui/ensembleLibraryEditor/tree/NumberClassNotFoundException.html (1.1)
- gui/ensembleLibraryEditor/tree/NumberNode.html (1.1)
- gui/ensembleLibraryEditor/tree/NumberNodeEditor.html (1.1)
- gui/ensembleLibraryEditor/tree/PropertyNode.html (1.1)

2006-06-30 01:55 fracpete

```
EnsembleSelection contribution by Robert Jung (mrbobjung at gmail dot com) and David Michael (dam68 at cornell dot edu)
```

- gui/EnsembleLibraryEditor.html (1.1)
- gui/EnsembleSelectionLibraryEditor.html (1.1)

2006-06-30 01:54 fracpete

```
EnsembleSelection contribution by Robert Jung (mrbobjung at gmail dot com) and David Michael (dam68 at cornell dot edu)
```

- gui/ensembleLibraryEditor/AddModelsPanel.html (1.1)
- gui/ensembleLibraryEditor/DefaultModelsPanel.html (1.1)
- gui/ensembleLibraryEditor/LibrarySerialization.html (1.1)
- gui/ensembleLibraryEditor/ListModelsPanel.html (1.1)
- gui/ensembleLibraryEditor/LoadModelsPanel.html (1.1)
- gui/ensembleLibraryEditor/ModelList.html (1.1)

2006-06-30 01:53 fracpete

```
EnsembleSelection contribution by Robert Jung (mrbobjung at gmail dot com) and David Michael (dam68 at cornell dot edu)
```

- classifiers/EnsembleLibrary.html (1.1)
- classifiers/EnsembleLibraryModel.html (1.1)
- classifiers/EnsembleLibraryModelComparator.html (1.1)

2006-06-30 01:51 fracpete

```
EnsembleSelection contribution by Robert Jung (mrbobjung at gmail dot com) and David Michael (dam68 at cornell dot edu)
```

- classifiers/meta/ensembleSelection/DefaultModels.props (1.1)
- classifiers/meta/ensembleSelection/EnsembleMetricHelper.html (1.1)
- classifiers/meta/ensembleSelection/EnsembleModelMismatchException.html (1.1)
- classifiers/meta/ensembleSelection/EnsembleSelectionLibrary.html (1.1)
- classifiers/meta/ensembleSelection/EnsembleSelectionLibraryModel.html (1.1)
- classifiers/meta/ensembleSelection/large\_binary\_class.model.xml (1.1)

2006-06-30 01:51 fracpete

```
EnsembleSelection contribution by Robert Jung (mrbobjung at gmail dot com) and David Michael (dam68 at cornell dot edu)
```

- classifiers/meta/ensembleSelection/ModelBag.html (1.1)
- classifiers/meta/ensembleSelection/large\_multi\_class.model.xml (1.1)
- classifiers/meta/ensembleSelection/toylist.model.xml (1.1)

2006-06-28 22:17 fracpete

```
registering of editors for the GenericObjectEditor is now based on weka/gui/GUIEditors.props file, which makes it easier to register new classes
```

- gui/GenericObjectEditor.html (1.55)

2006-06-28 07:33 fracpete

```
added checkLowerCaseNames flag (for PostgreSQL) and checkForTable flag (used in KnowledgeFlow)
```

- experiment/DatabaseUtils.props (1.6)

2006-06-28 07:05 fracpete

```
added try-catch-block around the ResultSet.getObject call in getCells
```

- gui/sql/ResultSetHelper.html (1.2)

2006-06-22 01:04 fracpete

```
Remco's new SVM for regression implementation
```

- gui/GenericPropertiesCreator.excludes (1.3)

2006-06-21 22:46 fracpete

```
helper class for accessing properties via Inflection and a property path, e.g., Vote contains two classifiers, J48 and LinearRegression. Then "classifiers[1].ridge" accesses the second classifier in the meta classifier, which is LinearRegression, and returns the "ridge" property.
```

- core/PropertyPath.html (1.1)

2006-06-20 04:56 mhall

```
Now implements Serializable
```

- core/AttributeExpression.html (1.3)

2006-06-19 23:19 mhall

```
Fixed problem with setting cost values
```

- gui/CostMatrixEditor.html (1.10)

2006-06-19 01:49 fracpete

```
Remco's new SVM for regression implementation
```

- classifiers/functions/supportVector/RegOptimizer.html (1.1)
- classifiers/functions/supportVector/RegSMOImproved.html (1.1)

2006-06-15 03:40 mhall

```
Initial import
```

- core/converters/URLSourcedLoader.html (1.1)

2006-06-08 02:22 eibe

```
updated
```

- core/version.txt (1.2)

2006-06-07 22:36 fracpete

```
fixed Javadoc
splitOptions no longer throws an exception for escaped chars other than " and \, eg \n and \t
joinOptions uses now backQuoteChars - option can then be used again in splitOptions and/or on commandline/SimpleCLI
```

- core/Utils.html (1.53)
